# Use of a ventilator-associated pneumonia (VAP) bundle to decrease the VAP rate in Syria

**DOI:** 10.1186/cc10681

**Published:** 2012-03-20

**Authors:** R Alsadat, M Mazloum, A Alshamaa, A Dakkak, H Al-Bardan, M Eltayeb, A Marie, F Esber, O Naes, M Shama, I Betelmal, M Kherallah

**Affiliations:** 1 Al-Mouassat Hospital, Damascus, Syria; 2 General Assembly of Damascus Hospital, Damascus, Syria; 3 Al-Bassel Heart Institute, Damascus, Syria; 4 Ibn Alnafees Hospital, Damascus, Syria; 5 World Health Organization, Damascus, Syria; 6 King Faisal Specialist Hospital and Research Center, Riyadh, Saudi Arabia

## Introduction

Implementation of a ventilator-associated pneumonia (VAP) bundle as a performance improvement project in the critical care units for all mechanically ventilated patients aiming to decrease the VAP rates over the study period at four major teaching hospitals in Damascus.

## Methods

CDC criteria were used to define VAP. VAP rates were calculated based on occurrences per 1,000 ventilator days, VAP rates were monitored on a monthly basis throughout the project period. VAP bundle elements included elevation of the head of the bed to between 30 and 45°, daily sedation vacation, daily assessment of readiness to wean, peptic ulcer disease prophylaxis and deep venous thrombosis prophylaxis if not contraindicated. Each hospital formed a task force with a team leader, one or two physicians and one or two nurses. Education took place at an initial conference and a follow-up meeting for the implementation process and frequent staff education session in individual units. Compliance with the VAP bundle was considered based on the implementation of all elements of the bundle. Statistical Control Chart (SPC) was used to monitor the compliance with the individual bundle elements as well the bundle as a whole.

## Results

VAP bundle compliance rates were steadily increasing from 33 to 80% in Hospital 1, from 33 to 86% in Hospital 2 and from 83 to 100% in Hospital 3 during the study period. The VAP bundle was not applied in Hospital 4 and therefore no data were available. This correlated with a decrease in VAP rates from 30 to 6.4 per 1,000 ventilator days in Hospital 1, from 12 to 4.9 per 1,000 ventilator days in Hospital 3, whereas the VAP rate failed to decrease in Hospital 2 (despite better compliance) and it remained high around 33 per 1,000 ventilator days in Hospital 4 where the VAP bundle was not implemented or monitored.

## Conclusion

The VAP bundle is known to be an effective way to decrease VAP but has performed differently in different hospitals in our study. Prevention of VAP requires concerted efforts on the part of hospital administration, physicians, and ICU personnel. The program must be evidence-based, maintained, and accepted by ICU personnel. Monitoring and collection of data should be strict and objective. Continued education and feedback are crucial to maintain a low VAP rate. Other factors of healthcare infection prevention should also be taken into consideration.

